# Associations between apparent diffusion coefficient (ADC) and KI 67 in different tumors: a meta-analysis. Part 1: ADC_mean_

**DOI:** 10.18632/oncotarget.20406

**Published:** 2017-08-24

**Authors:** Alexey Surov, Hans Jonas Meyer, Andreas Wienke

**Affiliations:** ^1^ Department of Diagnostic and Interventional Radiology, University of Leipzig, Leipzig, Germany; ^2^ Institute of Medical Epidemiology, Biostatistics, and Informatics, Martin Luther University of Halle-Wittenberg, Halle, Germany

**Keywords:** diffusion weighted imaging, ADC, ki 67

## Abstract

Diffusion weighted imaging (DWI) is a magnetic resonance imaging (MRI) technique based on measure of water diffusion in tissues. This diffusion can be quantified by apparent diffusion coefficient (ADC). Some reports indicated that ADC can reflect tumor proliferation potential. The purpose of this meta-analysis was to provide evident data regarding associations between ADC and KI 67 in different tumors. Studies investigating the relationship between ADC and KI 67 in different tumors were identified.

MEDLINE library was screened for associations between ADC and KI 67 in different tumors up to April 2017. Overall, 42 studies with 2026 patients were identified. The following data were extracted from the literature: authors, year of publication, number of patients, tumor type, and correlation coefficients.

Associations between ADC and KI 67 were analyzed by Spearman's correlation coefficient. The reported Pearson correlation coefficients in some studies were converted into Spearman correlation coefficients.

The pooled correlation coefficient between ADC_mean_ and KI 67 for all included tumors was *ρ* = −0.44. Furthermore, correlation coefficient for every tumor entity was calculated. The calculated correlation coefficients were as follows: ovarian cancer: *ρ* = −0.62, urothelial carcinomas: *ρ* = −0.56, cerebral lymphoma: *ρ* = −0.55, neuroendocrine tumors: *ρ* = −0.52, glioma: *ρ* = −0.51, lung cancer: *ρ* = −0.50, prostatic cancer: *ρ* = −0.43, rectal cancer: *ρ* = −0.42, pituitary adenoma:*ρ* = −0.44, meningioma, *ρ* = −0.43, hepatocellular carcinoma: *ρ* = −0.37, breast cancer: *ρ* = −0.22.

## INTRODUCTION

Diffusion weighted imaging (DWI) is a magnetic resonance imaging (MRI) technique based on measure of water diffusion in tissues [1]. This diffusion can be quantified by apparent diffusion coefficient (ADC) [1, 2]. Furthermore, ADC can be divided into three sub-parameters: ADC minimum or ADC_min_, mean ADC or ADC_mean_ and ADC maximum or ADC_max_ [2]. Most frequently, ADC_mean_ is used in clinical and experimental investigations. Previously, numerous reports showed usefulness of DWI/ADC in oncology [3–8]. According to the literature, ADC values can discriminate malignant and benign lesions [7, 9]. Typically, malignant tumors have lower values in comparison to benign lesions [7, 9]. For example, in head and neck region, malignant lymphomas had a mean ADC value of 0.66 × 10^−3^ mm^2^s^−1^, squamous and adenoid carcinomas 1.13 × 10^−3^ mm^2^s^−1^, while benign solid lesions presented with a mean ADC value of 1.56 × 10^−3^ mm^2^s^−1^ [9].

Furthermore, previous studies also mentioned that ADC can predict early response to treatment and clinical outcome in different malignancies [3–7, 10, 11]. So Papaevangelou et al. demonstrated an early increase of ADC values under cytostatic therapy in experimental colonic cancer [13]. Histopathological examination identified thereby a decrease of vital cells [12]. Moreover, numerous clinical investigations of different tumors, for example, ovarian carcinomas [10], lung, esophageal, gastric, rectal cancer or liver metastases showed similar results [11, 13].

These effects of ADC are based on its associations with several histopathological features. It has been shown that ADC correlated inversely with cell count of investigated lesions [1, 2, 14]. However, as suggested in a recent meta-analysis, this correlation is different in several tumors [14]. Thereby, correlation coefficients ranged from *ρ* = −0.25 in lymphoma to *ρ* = −0.66 in glioma [14].

As mentioned by some authors, ADC can also reflect other histopathological features, such as expression of different receptors, nucleic polymorphism, and proliferation potential [2]. Especially associations with proliferation, for example, with expression of MIB 1 or KI 67 receptor are very important because the fact that it predicts behavior of several tumors [2, 15]. According to the literature, breast carcinomas with high expression of KI 67 had lower ADC values in comparison to tumors with low KI 67 expression [15]. Also in meningioma and cerebral lymphoma, ADC can distinguish between tumors with low and high expression of KI 67 [16, 17].

However, use of ADC as a biomarker of tumor proliferation is difficult because of several problems. Firstly, a wide spectrum of correlation coefficients between ADC and KI 67 was reported [17–59]. Secondly, most reports about associations between ADC and KI 67 investigated small samples ranging from 11 to 50 patients/tumors [17, 22–27]. There were only few studies investigated collectives over 100 patients [28–30].

The purpose of this meta-analysis was to provide evident data regarding associations between DWI, in particular ADC_mean_, and KI 67 in different tumors.

## RESULTS

The enrolled studies comprised 2026 patients with several tumors. Most frequently, different breast tumors (28.28%), followed by glioma (10.81%), urothelial carcinomas (10.41%), neuroendocrine tumors (9.53%), rectal cancer (7.75%), menigioma (5.43%), and hepatocellular carcinoma (5.13%) were reported (Table 1). Other tumors were rarer.

**Table 1:** Overview about all involved tumor types

Diagnosis	*n*	%
Different breast tumors and tumor like lesions	573	28.28
Glioma	219	10.81
Urothelial carcinoma	211	10.41
Neuroendocrine tumor	193	9.53
Rectal cancer	157	7.75
Meningioma	110	5.43
Hepatocellular carcinoma	104	5.13
Ovarian tumor	86	4.25
Prostatic cancer	81	4.00
Lung cancer	51	2.52
Cerebral lymphoma	49	2.42
Pituary adenoma	41	2.02
Brain metastases	32	1.58
Pancreatic cancer	28	1.38
Different brain tumors	26	1.28
Uterine cervical cancer	21	1.04
Liver metastases	19	0.94
Thyroid cancer	14	0.69
Head and neck cancer	11	0.54
**Total**	**2026**	**100**

**Figure 1:**
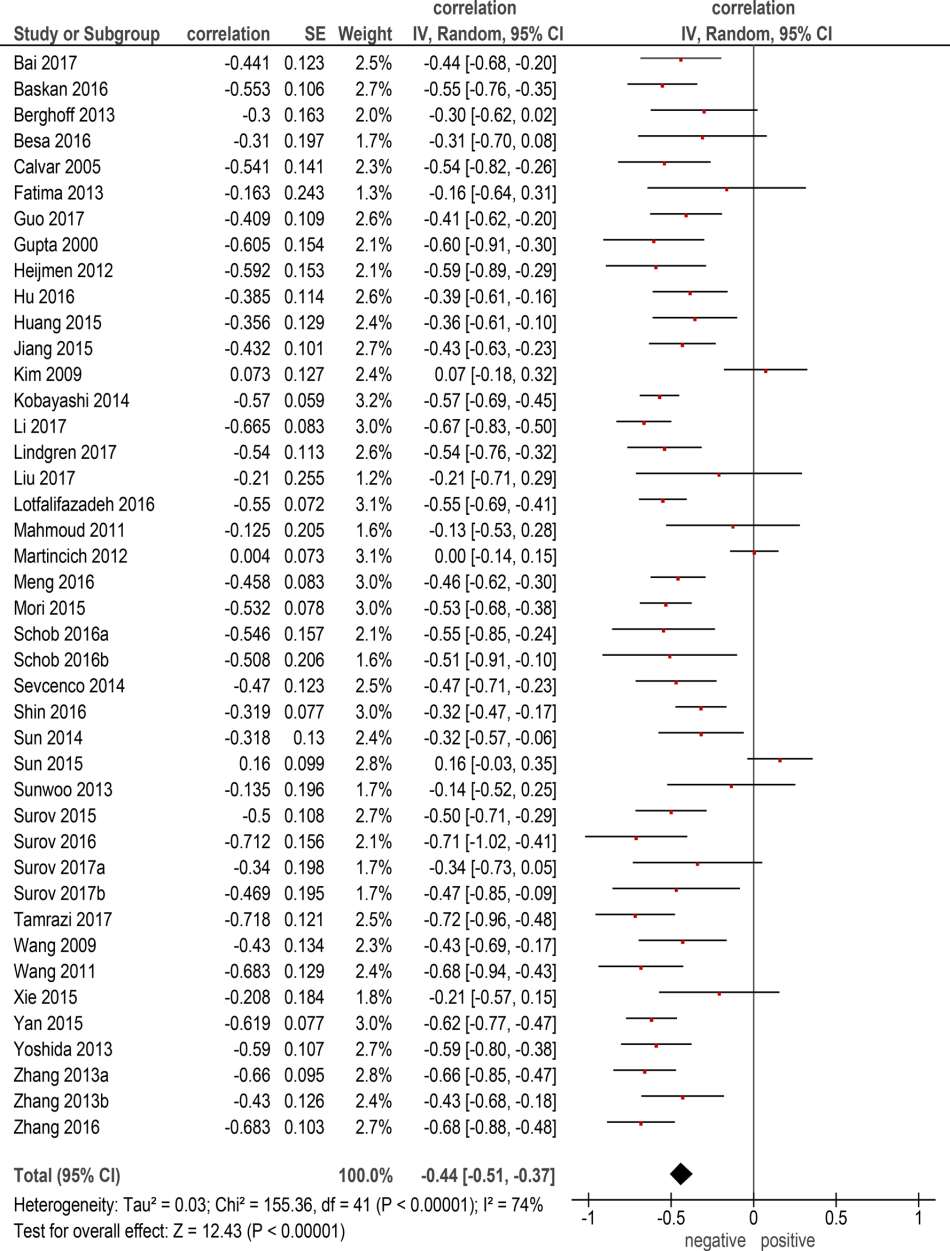
Forest plots of correlation coefficients between ADC_mean_ and KI 67 in all involved studies (*n* = 42)

The pooled correlation coefficient between ADC_mean_ and KI 67 for all included tumors (Figure 1) was *ρ* = −0.44, (95% CI = [−0.51;−0.37]), heterogeneity τ^2^ = 0.03, (*p* < 0.00001), I^2^ = 74 %, test for overall effect Z = 12.43 (*p* < 0.00001).

**Figure 2:**
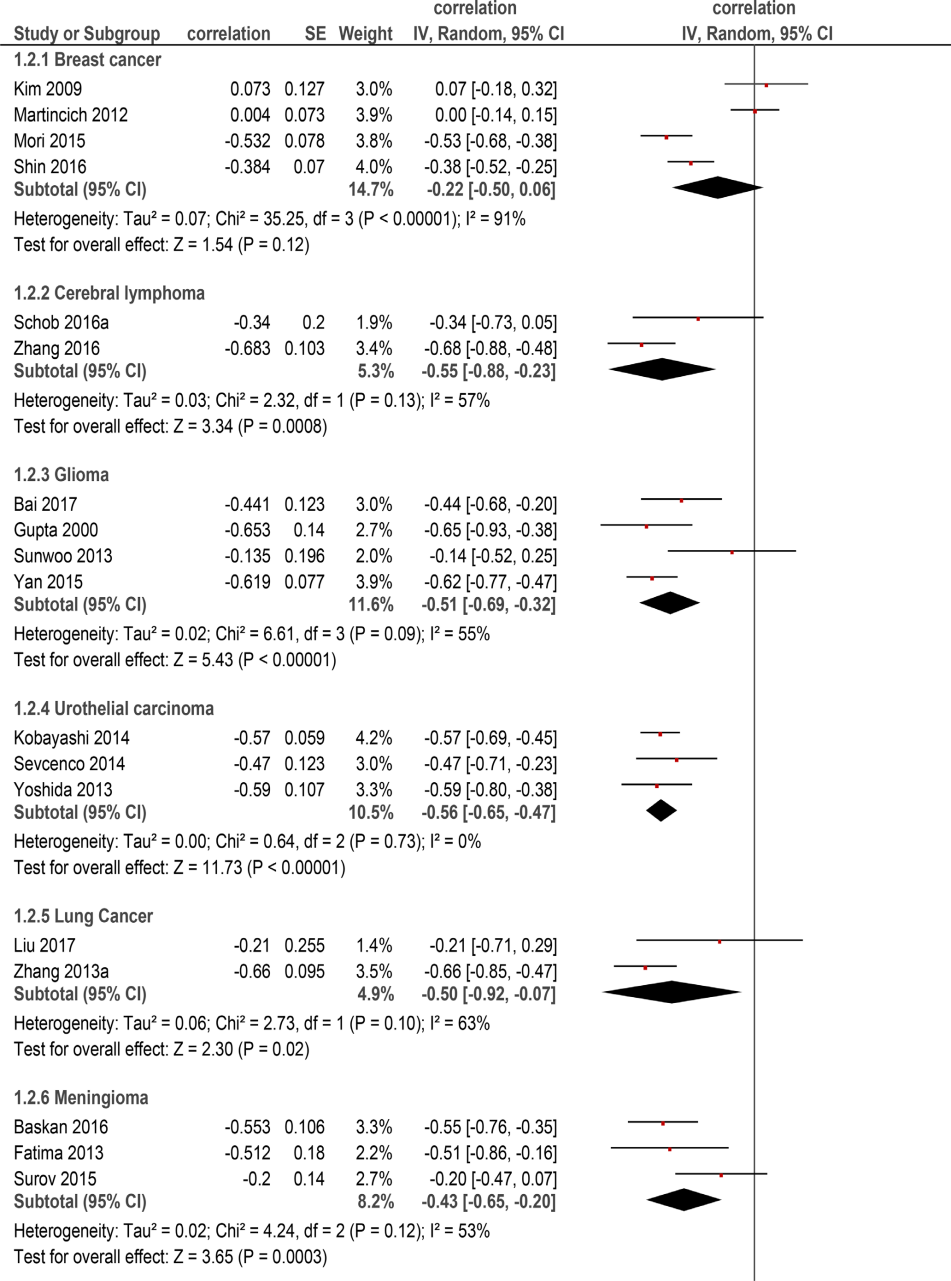

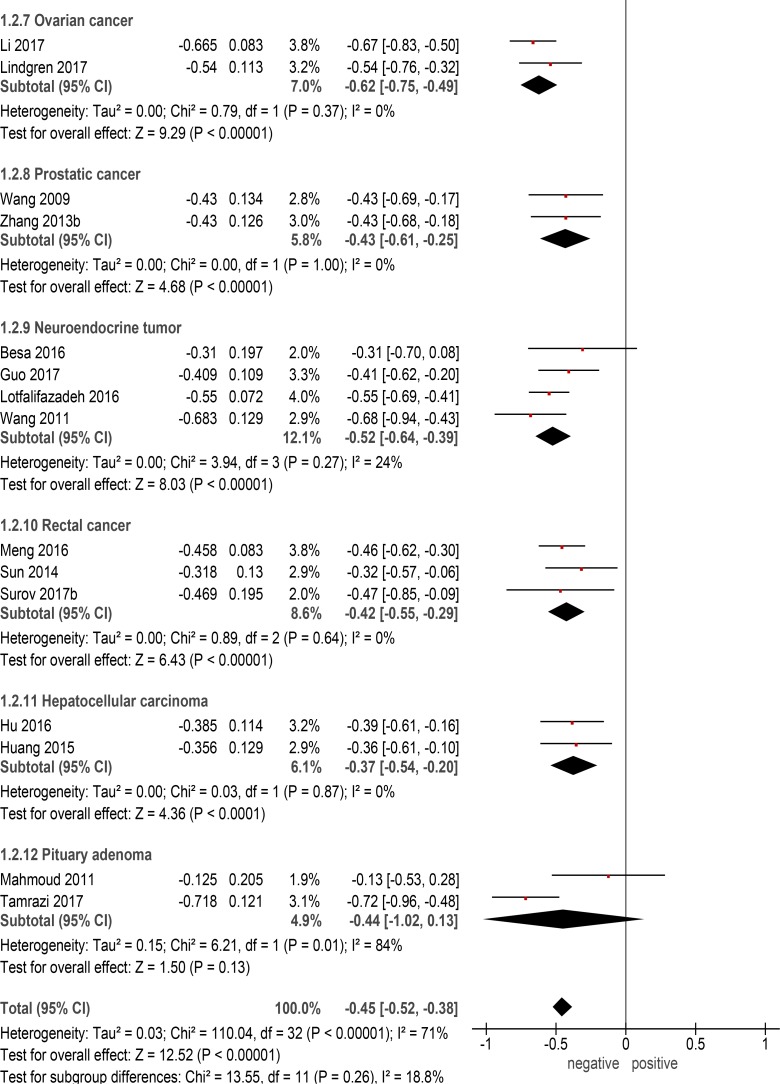
Forest plots of correlation coefficients between ADC_mean_ and KI 67 in different primary tumors

Furthermore, correlation coefficient for every tumor entity was calculated. For this sub-analysis, only data for primary tumors were acquired and tumor entities with less than three reports were excluded. Overall, 12 tumor entities with 1778 patients were included into the sub-analysis (Table 2). The calculated correlation coefficients were as follows: ovarian cancer: *ρ* = −0.62 (95% CI = [−0.75; −0.49]); urothelial carcinomas: *ρ* = −0.56 (95% CI = [−0.65; −0.47]); cerebral lymphoma: *ρ* = −0.55 (95% CI = [−0.88; −0.23]); neuroendocrine tumors: *ρ* = −0.52 (95% CI = [−0.64; −0.39]); glioma: *ρ* = −0.51 (95% CI = [−0.69; −0.32]); lung cancer: *ρ* = −0.50 (95% CI = [−0.92; −0.07]); prostatic cancer: *ρ* = −0.43 (95% CI = [−0.61; −0.25]); rectal cancer: *ρ* = −0.42 (95% CI = [−0.55; −0.29]); pituitary adenoma:*ρ* = −0.44 (95% CI = [−1.00; 0.13]); meningioma, *ρ* = −0.43 (95% CI = [−0.65; −0.20]); hepatocellular carcinoma: *ρ* = −0.37 (95% CI = [−0.54; −0.20]); breast cancer: *ρ* = −0.22 (95% CI = [−0.50; 0.06]) (Figure 2).

**Table 2:** Tumor entities included into the subgroup analysis

Diagnosis	*n*
Breast cancer	476
Glioma	219
Urothelial carcinoma	211
Neuroendocrine tumor	193
Rectal cancer	157
Meningioma	110
Hepatocellular carcinoma	104
Ovarian tumor	86
Prostatic cancer	81
Lung cancer	51
Cerebral lymphoma	49
Pituary adenoma	41
**Total**	**1778**

## DISCUSSION

To the best of our knowledge, this is the first meta-analysis regarding associations between ADC and KI 67 in different tumors based on a large sample. As seen, in the general collective, ADC correlates moderately with KI 67.

Some previous investigations identified the phenomenon that ADC can be associated with KI 67 [2, 17, 22, 24, 29, 56]. The exact cause of this association is unclear. KI 67 is a nonhistone, nuclear protein synthesized throughout the whole cell cycle except the G0 phase, and has been shown to be responsible for cell proliferation [60, 61]. It is well known that the nucleic size increases during mitosis [62]. Previous investigations identified statistically significant correlations between nucleic size/volume and ADC [2, 22, 51, 63]. Furthermore, intracellular water diffusion may be affected by numerous mitotic membranes and tubular structures [64]. It is also possible that mitotic phases may induce an increase of cytoplasmic proteins and, therefore, increase of cytoplasmic viscosity [65]. This may also decrease ADC.

Independent of possible pathomechanisms of interaction between ADC and KI 67, a key question is how ADC is helpful to predict proliferation potential of investigated tumors or not. Our analysis showed that the reported data about associations between ADC and KI 67 are very inconsistent. While some authors identified significant correlations between the parameters, other did not. Presumably, several tumors may show also different relationships between ADC and KI 67. In fact, our meta-analysis confirmed this hypothesis. In ovarian cancer, ADC correlated well with KI 67. This finding suggests the possibility to use ADC as a biomarker for proliferation in this tumor. In most investigated tumors, such as in urothelial carcinoma, lung cancer, cerebral lymphoma, and neuroendocrine tumors ADC correlated moderately with KI 67 and the correlation coefficients ranged from −0.50 to −0.56. Hence, we postulate that ADC may be used as an additional surrogate marker for proliferation potential for these tumors, however, his validity is restricted.

Furthermore, weak-to-moderate correlations between ADC and KI 67 were identified in meningiomas, rectal cancer, prostatic cancer, and pituitary adenomas. In addition, in breast cancer and hepatocellular carcinoma, weak correlations between ADC and KI 67 were found. Therefore, ADC cannot be used as a proliferation biomarker in these entities.

Interestingly, the present data are almost concordant with those reported for associations between ADC and cell count in several tumors [14]. It has been shown that ADC correlated strongly with cell count in gliomas, ovarian cancer, and lung cancer [14]. Moderate correlations were identified between ADC and cell count in prostatic cancer, renal cell carcinoma, uterine cervical cancer, and head/neck squamous cell carcinomas [14]. Finally, weak-to-moderate correlations were found in breast cancer and meningioma and weak correlation was identified in lymphomas [14]. This finding suggests that relationships between ADC and KI 67 as well with cell count are similar.

Beside the mentioned results, several problems were identified, which limited our meta-analysis. Firstly, there are only 12 tumor entities, which were involved into the work. Furthermore, only 7 entities, namely breast cancer, glioma, urothelial carcinoma, neuroendocrine tumors, rectal cancer, and meningioma contained relatively large patient samples ranging from 104 to 476. In addition, as seen, significant heterogeneities among the studies for the same tumors were identified. For example, in the breast cancer, Kim et al. reported the correlation of 0.07, but in the study of Mori et al. it was −0.53. This finding is difficult to ascertain. These variations of the published correlation coefficients were possibly due to different population of subjects, different ratio of tumor subtypes, or different method of analysis (ROI size, location, etc.). Clearly, the results of the present meta-analysis may be limited due this fact.

For other tumors, such as ovarian cancer, prostatic cancer, lung cancer, cerebral lymphoma, and pituitary adenoma, the number of patients was small ranging from 41 to 86. This fact relativizes the validity of the estimated correlation coefficients.

Secondly, only one report was published for pancreatic carcinomas, thyroid cancer, head and neck squamous cell carcinoma, and uterine cervical cancer, respectively. Therefore, these tumors could not be included into the subgroups analysis.

Furthermore, we identified another great problem. To date, there are no reports about relationships between ADC and KI 67 in frequent and less frequent solid malignancies, such as colonic cancer, esophageal carcinoma, gastric cancer, gastrointestinal stromal tumors, renal cell carcinoma, different sarcomas, pleural and peritoneal mesotheliomas, thymic cancer, gall bladder cancer, and adrenal gland carcinoma. This is a purpose for further investigations.

In conclusion, several tumors showed different inverse correlations between ADC and KI 67. Strong correlation was found in ovarian cancer, and, therefore, ADC can be used as an imaging marker for proliferation potential in this entity.

In urothelial carcinoma, lung cancer, cerebral lymphoma, glioma,and neuroendocrine tumors moderate correlations were identified between ADC and KI 67. Therefore, use of ADC as a surrogate marker for proliferation potential in clinical practice is limited.

In meningiomas, rectal cancer, prostatic cancer, and pituitary adenomas, weak-to-moderate correlations and in breast cancer and hepatocellular carcinoma, weak correlations between ADC and KI 67 were found. This finding indicates that ADC cannot predict proliferation potential in these entities.

Finally, for other tumors, no evident data can be provided to date.

## MATERIALS AND METHODS

### Data acquisition and proving

**Figure 3:**
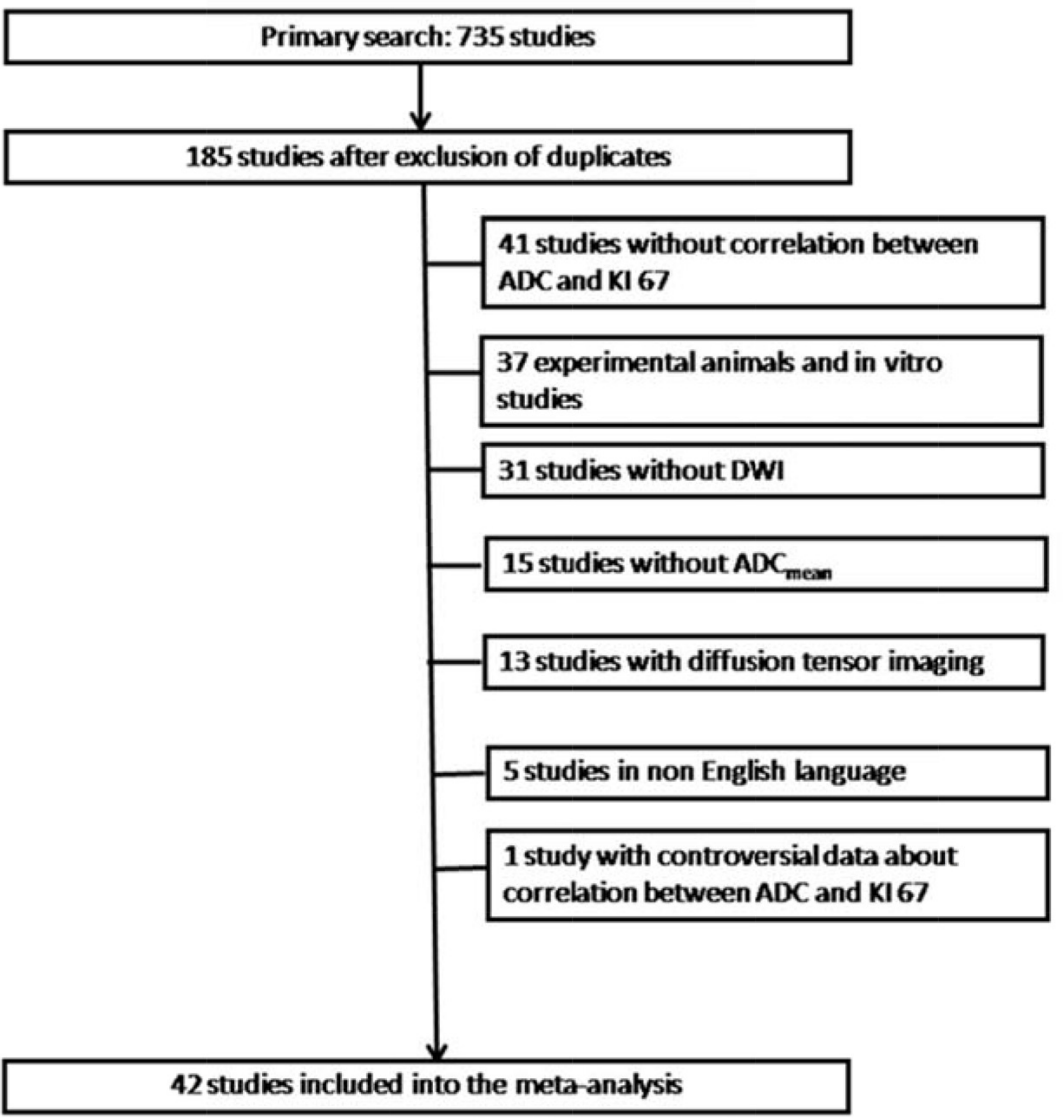
Flowchart of the study selection

The strategy of data acquisition is shown in Figure 3. MEDLINE library was screened for associations between ADC and KI 67 in different tumors up to April 2017. The following search words were used: “DWI or diffusion weighted imaging or diffusion-weighted imaging or ADC or apparent diffusion coefficient AND KI 67 OR KI67 OR ki67 OR ki-67 OR mitotic index OR proliferation index OR MIB 1 OR MIB-1 OR mitosis index”. Secondary references were also recruited. The Preferred Reporting Items for Systematic Reviews and Meta-Analyses statement (PRISMA) was used for the research [66].

Overall, 735 records were identified. After exclusion of duplicates (*n* = 550), a total of 185 publications were included into the further analysis. For this work, only data regarding ADC_mean_ derived from diffusion weighted imaging (DWI) were collected. Overall, 143 publications were excluded. There were 31 studies without DWI, 5 non English publications, 41 publications, which did not contain correlation coefficients between ADC and KI 67, and 37 experimental animals and *in vitro* studies. Furthermore, data retrieved from diffusion tensor imaging and studies with other than ADC_mean_ parameters were also excluded (*n* = 28). Finally, we excluded one study with wrong data regarding correlation coefficient between ADC and KI 67. Therefore, the present analysis comprises 42 studies with 2026 patients [17–59]. The following data were extracted from the literature: authors, year of publication, number of patients, tumor type, and correlation coefficients.

### Meta-analysis

The methodological quality of the 42 studies was independently checked by two observers (A.S. and H.J.M.) using the Quality Assessment of Diagnostic Studies (QUADAS) instrument according to previous descriptions [67, 68]. Table 3 shows the results of QUADAS proving.

**Table 3:** Methodological quality of the involved 42 studies according to the QUADAS criteria

QUADAS criteria	yes (%)	no (%)	unclear (%)
Patient spectrum	40 (95.24)		2 (4.76)
Selection criteria	29 (69.05)	12 (28.57)	1 (2.38)
Reference standard	42 (100)		
Disease progression bias	42 (100)		
Partial vertification bias	42 (100)		
Differential vertification bias	42 (100)		
Incorporation bias	42 (100)		
Text details	42 (100)		
Reference standard details	42 (100)		
Text review details	18 (42.86)	10 (23.81)	14 (33.33)
Diagnostic review bias	20 (47.62)	10 (23.81)	12 (28.57)
Clinical review bias	40 (95.24)	1 (2.38)	1 (2.38)
Uninterpretable results	42 (100)		
Withdrawls explained	40 (95.24)		2 (4.76)

Associations between ADC_mean_ and KI 67 were analyzed by Spearman’s correlation coefficient. The reported Pearson correlation coefficients in some studies were converted into Spearman correlation coefficients according to the previous description [69].

Furthermore, the meta-analysis was undertaken by using RevMan 5.3 (*Computer program, version 5.3. Copenhagen: The Nordic Cochrane Centre, The Cochrane Collaboration, 2014*)*.* Heterogeneity was calculated by means of the inconsistency index I^2^ [70, 71]. In a subgroup analysis, studies were stratified by tumor type. In addition, DerSimonian and Laird random-effects models with inverse-variance weights were used without any further correction [72].
